# Psychometric properties of the National Eye Institute Visual Function Questionnaire (NEI-VFQ 25) in a Norwegian population of patients with neovascular age-related macular degeneration compared to a control population

**DOI:** 10.1186/s12955-019-1203-0

**Published:** 2019-08-14

**Authors:** Elma Jelin, Torbjørn Wisløff, Morten C. Moe, Turid Heiberg

**Affiliations:** 1 Department of Ophthalmology, Oslo University Hospital, Postboks 4950 Nydalen, 0424 Oslo, Norway; 2 Institute of Clinical Medicine, University of Oslo, Postboks 1171 Blindern, 0318 Oslo, Norway; 3 Norwegian Institute of Public Health, Postboks 222 Skøyen, 0213 Oslo, Norway; 4 Institute of Health and Society, University of Oslo, Oslo, Norway; 5 Department of Regional Research Support, Oslo University Hospital, Sogn Arena, POBox 4950, Nydalen N-0424 Oslo, Norway; 6 Faculty of Health and Welfare, Østfold University College, POBox 700, N-1757 Halden, Norway

**Keywords:** Age-related macular degeneration, Intravitreal injections, Anti-VEGF, Patient reported outcome measures, NEI-VFQ 25, Validation, Questionnaire

## Abstract

**Background:**

Although visual acuity and optical coherence tomography (OCT) are most widely used as outcomes in treatment of neovascular age-related Macular Degeneration (nAMD), patient reported outcome measures are increasingly recognized. National Eye Institute Visual Function Questionnaire (NEI-VFQ 25) was developed to capture the perceived visual function. Yet, evidence of psychometric performance in the target population is required. The aim of this study was to examine the psychometric properties of NEI-VFQ 25 in a Norwegian cohort of newly diagnosed nAMD patients followed with a Treat and Extend (T/E) protocol.

**Methods:**

Patients receiving intravitreal anti–vascular endothelial growth factor (anti-VEGF) injection treatment according to a T/E protocol completed a Norwegian translation of NEI-VFQ 25, EuroQoL Health Questionnaire (EQ-5D), and Patient acceptable symptom state (PASS 5) at baseline, 3, 6 and 12 months. In addition, a control population completed the same questionnaires. Visual acuity was assessed with LogMar for best/treated eye. Validity testing comprised face validity by a 0–10 numeric rating scale about relevance of NEI-VFQ 25 as well as regression analyses and correlations between NEI-VFQ 25 and other relevant variables. Reliability was examined with Intraclass Correlation Coefficient (ICC) and Cronbach’s alpha for internal consistency were performed. Responsiveness, discriminatory power and predictive value were also explored.

**Results:**

Number of respondents at baseline, after 3, 6 and 12 months was 197, 186, 176 and 168, respectively. The control population comprised 26 individuals. Face validity of NEI-VFQ 25 had a mean (SD) of 7.8 (1.7) (*n* = 84). NEI-VFQ was significantly correlated to visual acuity and PASS 5 as well as EQ-5D at baseline. Reliability (ICC) of the overall and sub scores for the patients/controls ranged from 0.49–0.97/0.59–0.97. Cronbach’s alpha was 0.61–0.85. Discriminatory power was confirmed by significant differences of the overall score between controls and patients (*P* < 0.001). NEI-VFQ 25 indicates responsiveness showing overall score improved significantly (*P* ≤ 0.001) from baseline to 3 months. NEI-VFQ 25, general health and visual acuity at baseline were the strongest predictors for how patients reported vision after 6 months follow-up.

**Conclusion:**

NEI-VFQ 25 showed acceptable psychometric performance, which supports that the Norwegian version can be used to monitor patients treated for nAMD.

## Introduction

Visual acuity levels and retinal thickness measured with optical coherence tomography (OCT) are often used as primary outcome measures in evaluating treatment for neovascular age-related Macular Degeneration (nAMD) [1, 2]. However, it is increasingly important to reflect the patient’s perspective concerning not only the treatment effect, but also the visual function in everyday life [3–6]. The Food and Drug Administration (FDA) and The European Medicines Agency (EMA) recommend researchers to show benefit in patient reported outcome measures (PROMs) for approval of new drugs or treatments [7–9]. Generic PROMs are useful in comparisons across diseases and populations, while disease-specific PROMs are usually more sensitive to disease-related characteristics. The two kinds of PROMs are recommended to be used in conjunction [10].

NEI-VFQ 25 is a vision specific PROM, reporting on visual function in everyday life [4, 11, 12]. NEI-VFQ 25 has been validated across ocular diseases such as glaucoma, cataract and AMD in different countries across the world including Sweden, Greece, Denmark, Serbia, Turkey, Germany, Italy, China, Thailand, Japan, and Brazil [13–18]. NEI-VFQ 25 has also been shown to correlate with visual acuity in patients treated with repetitive intravitreal anti-VEGF injections for nAMD [19, 20] and is considered as a tool which is sensitive to change in visual acuity [5, 19, 21]. Psychometric testing of a questionnaire is always required in target population and a new cultural setting [22, 23]. The aim of this study was to test psychometric performance of the Norwegian version of NEI-VFQ 25 in a longitudinal cohort study of newly diagnosed nAMD patients treated with an individualized Treat and Extend (T/E) protocol.

## Methods

### Patients and controls

Newly diagnosed nAMD patients with indication for intravitreal anti-VEGF therapy, as well as controls without known eye disease above 60 years of age were included in the study. The standard anti-VEGF protocol used was an individualized T/E regimen with bevacizumab as first-line of treatment as previously described [24–26]. The controls were relatives following patients to their clinical examination at the hospital. For both groups, we included Scandinavian speaking persons without cognitive impairment. Recruitment procedure was performed by researchers who were not involved in diagnosis or treatment.

### Data collection

The participants who met the inclusion criteria completed the baseline questionnaire at the outpatient clinic. Patients were interviewed at baseline, as well as 3, 6 and 12 months after the initiation of treatment. A subset of 30 patients to examine test-retest reliability was interviewed one week after 6 months. The controls responded to the questionnaire initially at the outpatient clinic and then one week after for test-retest. All follow-up interviews were performed by the telephone.

### NEI-VFQ 25

Both patients and controls responded to NEI-VFQ 25, which measures patient reported visual function. NEI-VFQ 25 contains 25 questions on visual function in daily life. This 25-item questionnaire is a shorter version of an original questionnaire containing 51 questions. There are also other versions of NEI-VFQ [27].

NEI-VFQ 25 has a total score ranging from 0 to 100 (where 100 reflects best health). It contains 12 subcategories: general health, general vision, ocular pain, near vision activities, distance activities, social functioning, mental health, role difficulties, dependency, driving, color vision and peripheral vision. The interviewer-administered format of NEI-VFQ 25 was used. The interviewer-administrated version involves interviewer reading the questions to the patients and writing down the patient’s answers. This applied version of NEI-VFQ 25 was translated to Norwegian by Mapi Trust (Mapi Trust 27 rue de la Villette, 69003 Lyon, France), which also gave permission to use the Norwegian version. Mapi Trust did a linguistic validation process which was officially approved by the developer of the original instrument.

### Other instruments

To be able to compare NEI-VFQ 25 with other relevant instruments, patients responded to EQ-5D-3 L EuroQoL Health Questionnaire 3 Level version [28] to measure utility. EQ-5D-3 L contains 5 questions on mobility, self-care, usual activities, pain/discomfort and anxiety/depression with 3 graded response options. We also used PASS 5 (Patient Acceptable Symptom Status) [29], which can be used as a measure for the level of acceptability of symptom status on a Likert scale 1–5 (where 1 is best, and 3 is acceptable) in this case visual symptom state.

Visual acuity was measured as the Logarithm of the minimal angle of resolution (LogMar) for both eyes from routine measurements at consultations in the outpatient clinic. Demographic data were collected to describe the age, gender, employment and marital status of the study population. All data collection for PROMs was performed as interviews due to the patient’s poor vision and high age.

### Analyses

Face validity was tested in a subgroup of 84 patients who graded the relevance of the questionnaire on a numeric rating scale from 0 to 10, where 10 indicated high relevance.

Unadjusted and adjusted regression analyses were used to identify associations between NEI-VFQ 25 overall score and EQ-5D, PASS 5, demographic variables, as well as clinical variables like acuity of best and treated eye in LogMar. Further, we did correlation analyses which also included sub scores of NEI-VFQ 25. Q-Q plots and median versus mean values indicate that the data were mainly not normally distributed. We therefore chose to use Spearman correlation analyses.

Test-retest reliability was examined one week apart at 6 months follow-up for patients and one week after baseline for the controls with Intraclass Correlation Coefficient (ICC) 2 way-mix method. Reliability was examined both for the overall and the sub-scores which were based on more than one item. ICC was evaluated to be either poor (< 0.5), moderate (0.5–0.75), good (0.75–0.9) or excellent (> 0.9) according to criteria described by Koo and Li [30]. Internal consistency was tested using Cronbach’s alpha on the overall and sub scores of NEI-VFQ 25.

Paired T-tests (baseline vs 3 months) were performed to examine responsiveness of NEI-VFQ 25. The same tests were performed on LogMar treated and best eye. Two-sample T-tests were used for the testing the discriminatory power of NEI-VFQ 25 between the patient and control groups.

Finally, the predictive value of baseline NEI-VFQ 25 was examined by a multivariable regression analysis with the overall score at 6 months as the dependent variable. The multivariable regression analysis included relevant variables as EQ-5D, PASS 5, demographic variables, and clinical variables like acuity of best and treated eye in LogMar.

## Results

Newly diagnosed nAMD patients (71% females, mean (SD) age 83.4 (7.6) years) responded to NEI-VFQ 25 at baseline (*n* = 197), 3 (*n* = 186), 6 (*n* = 176) and 12 (*n* = 168) months after initiating anti-VEGF treatment. The mean number of injections during 3 months was 2.35 (0.85) and 4.31 (1.61) during the 6 months follow-up. The majority of patients were treated with bevacizumab (97% at baseline). Significant improvement at 3, 6 and 12 months was found for LogMar in best (*P* < 0.05) and treated eye (*P* < 0.001). In addition, 26 controls (48% females, mean (SD) age 70.2 (7.7) years) were included in the study.

The mean overall score of NEI-VFQ 25 at baseline was 79.5 and 91.3 out of 100 in patients and controls, respectively. The sub-scores in patients varied from 46.8 (general health) to 94.4 (peripheral and color vision). Controls had a variation in sub-scores from 65.4 (general health) to 99.0 (color vision). Visual acuity in patients was 20/31 for best and 20/78 for treated eye (LogMar 0.19/0.59). Self-reported level of acceptable (vision) symptom status (PASS 5) was reported acceptable or beyond (54.3%) in the patient group, while 92% in the control group reported acceptable level or beyond (Table 1).

**Table 1:** Baseline descriptive characteristics

	Patients (*N* = 197)	Controls (*N* = 26)
Age (years)	83.4 (7.6)	70.2 (7.7)
Females	71%	48%
Married	44%	83.3%
Retired	91%	60%
Visual Acuity of best eye (LogMar)	0.19 (0.25)	NA
Fractional	20/31	
Visual Acuity of treated eye (LogMar)	0.59 (0.51)	NA
Fractional	20/78	
NEI-VFQ 25 overall score of 100	79.5 (14.5)	91.3 (7.7)
NEI-VFQ 25: sub scores:		
General Health	46.8 (23.1)	65.4 (25.6)
General vision	57.1 (17.7)	83.1 (12.3)
Ocular Pain	88.4 (19.5)	85.0 (18.7)
Near activities	74.1 (22.4)	88.0 (13.0)
Distance activities	75.7 (24.6)	91.7 (9.0)
Social Function	85.1 (21.6)	98.0 (6.0)
Mental health	77.2 (19.0)	91.0 (10.4)
Role difficulties	69.3 (30.0)	90.0 (20.1)
Dependency	89.7 (16.5)	98.7 (5.2)
Driving	61.9 (38.0)	83.7 (20.0)
Color vision	94.4 (14.1)	99.0 (5.0)
Peripheral vision	94.4 (14.5)	95.2 (10.4)
EQ-5D index	0.74 (0.28)^a^	0.85 (0.18)
PASS 5- Visual symptom status:		
Acceptable and over	54.3%	92.0%
Below acceptable	45.6%	8.0%

*NA* = Not available in this group. Available in *N* = 132

### Validity of NEI-VFQ 25

Face validity of NEI-VFQ 25 showed a mean (SD) of 7.8 (1.7) for the numeric rating scale.

NEI-VFQ 25 overall score was not associated with demographic data, but significant associations were found between visual acuity at both best (*P* < 0.001) and treated eye (*P* = 0.01) at baseline as well as with PASS 5 and EQ-5D, both in unadjusted and adjusted multiple regression analyses (Table 2).

**Table 2:** Associations between demographic and disease related variables with NEI-VFQ 25 at baseline

Variable	Unadjusted effect	95% CI	*P*-value	Adjusted effect*	95% CI	*P*-value
Age	− 0.106	(− 0.374, 0.161)	0.44	0.15	(−0.08, 0.38)	0.21
Gender	3,056	(−1.410, 7.522)	0.18	1.84	(−1.89, 5.58)	0.33
Marital status	−0.546	(−2.003, 0.911)	0.46	−0.11	(−1.17, 1.39)	0.86
LogMar best eye	−26.367	(−33.321, −19.413)	< **0.001**	− 20.25	(− 26.76,-13.75)	< **0.001**
LogMar treated eye	− 7.258	(− 11.151, − 3.365)	< **0.001**	− 4.25	(− 7.46,-1.05)	**0.01**
EQ-5D score	19.439	(12.569, 26.309)	< **0.001**	17.83	(11.99, 23.66)	< **0.001**
PASS 5	−7.196	(−9.545, −4.846)	< **0.001**	−5.11	(− 7.15, − 3.07)	< **0.001**

*Adjusted for all variables in multiple regression analyses. Bold values = *P* < 0.001 and *P* < 0.05

The results from cross-sectional multiple regression analyses at 3-, 6- and 12-month follow-up were similar with regard to associations between NEI-VFQ 25 and demographic and disease related variables. However, NEI-VFQ 25 overall score was not significantly associated to EQ-D5 at 6 and 12 months and to LogMar treated eye at 12 months (data not shown).

Baseline sub-scores of NEI-VFQ 25 correlated significantly with visual acuity in both best and treated eye and with PASS 5, except for general health, ocular pain, and peripheral vision. General vision was not correlated to LogMar treated eye. EQ-5D was strongly correlated to general health sub-score but was not significantly correlated to color nor peripheral vision (more details in Table 3).

**Table 3:** Baseline correlations (Spearman) between visual acuity in LogMar treated- and best eye with NEI VFQ-25 Scores

VFQ-25 Subscale/ Overall Score	LogMar best eye	LogMar treated eye	EQ-5D	PASS
General health	− 0.10	0.33	0.48**	−0.25
General vision	−0.35**	−0.14	0.25**	−0.57**
Ocular pain	−0.02	0.08	0.18*	−0.05
Color vision	−0.23 **	−0.22**	0.05	−0.09
Near activities	−0.41**	−0.14*	0.26**	−0.40**
Distance activities	−0.31**	−0.15*	0.32**	−0.25**
Social function	−0.25**	−0.11	0.27**	−0.29**
Mental health	−0.32**	−0.20**	0.36**	−0.32**
Role difficulties	−0.30**	−0.16*	0.31**	−0.38**
Dependency	−0.35**	−0.20**	0.31**	−0.26**
Peripheral vision	−0.09	−0.15*	0.09	−0.14*
Driving	−0.37**	−0.29**	0.13	−0.24*
Total score	−0.40**	−0.21**	0.35**	−0.45**

Correlation coefficient *P < 0.05 **P < 0.001

### Reliability and internal consistency

ICC values were 0.91 for NEI-VFQ 25 overall score in the patient group and 0.96 in the control group. ICC values for all sub scores of NEI-VFQ 25 in the patient group were ranging from 0.49 for role difficulties to 0.97 for driving (Table 4). In the control group all sub scores except one showed an ICC value of 0.59 or higher (Table 4). There were 9 of 12 sub-categories with a > 0.7 ICC value in the patient group (Table 4). Concerning the internal consistency, the overall scores in Cronbach’s alpha was 0.85 for overall score and 0.61–0.78 in sub-scores [31].

**Table 4:** Internal consistency at baseline and test-retest reliability of NEI-VFQ 25 in patient and control group

NEI-VFQ 25	Number of items	Cronbach’s Alpha in patients (*N* = 175)	ICC in patients (*N* = 31)	ICC in controls (*N* = 21)
General Health	1	NA	0.64	0.78
General vision	1	NA	0.51	0.72
Ocular Pain	2	0.64	0.91	0.89
Near activities	3	0.74	0.87	0.65
Distance activities	3	0.77	0.90	0.68
Social Function	2	0.66	0.93	0.83
Mental health	4	0.69	0.80	0.65
Role difficulties	2	0.78	0.49	0.92
Dependency	3	0.76	0.75	0.59
Driving	2	0.61	0.97	0.97
Color vision	1	NA	0.89	−0.11
Peripheral vision	1	NA	0.96	0.60
25-item composite	25	0.85	0.91	0.96

*NA* = Not Applicable

*ICC* = Intraclass Correlation Coefficient

### Responsiveness, discriminatory power and predictive value

NEI-VFQ 25 overall score showed a significant improvement (P ≤0.001, CI:-3.55,-1.33 ) from baseline to 3 months (Table 5), that was also seen at 6 months (*P* = 0.001, CI:-3.88,-1.04), and at 12 months (*P* = 0.033, CI: − 3.33, − 0.14). Longitudinal changes were also statistically significant for PASS but not for EQ-5D (data not shown for 6 and 12 months).

**Table 5:** Improvement in NEI-VFQ 25 and other measures in nAMD patients after 3 months of intravitreal anti-VEGF treatment

Variables	Baseline	3 months	*P*-value of baseline-3 months
NEI-VFQ 25 score	79.53 (14.52)	82.20 (13.68)	**< 0.001**
EQ-5D score	0.74 (0.28)	0.70 (0.32)	0.64
PASS 5	3.29 (0.80)	2.82 (0.80)	**< 0.001**
LogMar best eye	0.19 (0.03)	0.15 (0.22)	**0.015**
LogMar treated eye	0.59 (0.49)	0.44 (0.42)	**< 0.001**

Bold values = *P* < 0.001 *P* < 0.05

NEI-VFQ 25 overall score was significantly different in nAMD patients and controls (*P* < 0.001 and CI:-17.639,-5.975), supporting discriminatory power. All sub scores also showed significant difference between the two groups, except for peripheral vision, color–vision and ocular pain (data not shown).

Baseline predictors of NEI-VFQ 25 overall score after 6 months were explored in a multivariable regression analysis. The following variables were significant; NEI-VFQ 25 (*P* = 0.001, CI = 0.53, 0.84), LogMar best eye (*P* = 0.02, CI = -19.05,-1.74) and EQ-5D score (*P* = 0.03, CI = 0.68, 15.27). LogMar treated eye was borderline significant (*P* = 0.05, CI = -8.99, 0.06) (Table 6). These results show that baseline NEI-VFQ 25 overall score, general health and visual acuity are the strongest predictors for how patients report vision after 6 months follow up.

**Table 6:** Baseline predictors of NEI-VFQ 25 overall score after 6 months explored in multivariable regression analyses

Variable	Unadjusted effect	95% CI	*P*-value	Adjusted effect*	95% CI	*P*-value
Age	−0.24	−0.52,0.05	0.11	0.09	−0.18,0.36	0.52
Gender	0.41	−0.34,5.16	0.86	−2.54	−7.19,2.11	0.28
Marital status	−1.05	−2.59,0.48	0.24	−0.88	−2.34,0.59	0.24
LogMar best eye	−25.58	−33.02, −18.14	< **0.001**	−10.40	−19.04,-1.74	**0.02**
LogMar treated eye	−6.87	−11.64,-2.10	**0.005**	−4.47	−8.99,0.06	**0.05**
EQ-5D score	18.01	8.41,27.61	< **0.001**	7.98	0.68,15.27	**0.03**
NEI-VFQ 25 baseline	0.80	0.71,0.90	< **0.001**	0.69	0.53,0.84	< **0.001**
Treatment intensity	1.53	−3.07,0.01	**0.05**	−0.47	−2.29,1.35	0.61
PASS 5	−4.83	−7.46,- 2.21	< **0.001**	0.15	−2.24,2.55	0.89

*Adjusted for all variables in multiple regression analyses. Bold values = *P* < 0.001 and *P* < 0.05

## Discussion

NEI-VFQ 25 was originally designed and validated for the American language and culture. The aim of this study was to test the psychometric properties of NEI-VFQ 25 in a Norwegian population of nAMD patients treated with intravitreal injections according to a T/E protocol. The longitudinal data in this study provided an opportunity to look at a broader set of analyses, like reliability, responsiveness and predictive value.

The added value of PROMs to clinical outcomes gives a better understanding of how treatment and disease is experienced by the patients [32]. Including EQ-5D for the validation of NEI-VFQ 25 was chosen because it is a valid, generic measure with the advantage of its brevity [33–35].

Including controls enabled us to test discriminatory power. However, a limitation to this is the relatively small sample size in the control group. We were unable to include sufficiently matched controls as to demographic characteristics since the mean age difference was 13 years between the patients and the controls and the sex ratio also differed. However, such differences are also seen in similar relevant studies [17, 36].

Using interviewer administered version of NEI-VFQ 25 made it possible to explain questions that were unclear for the patients who generally had high age and poor vision. However, the interviewer administered format can contain a risk of bias. Previous studies have shown that patients report better scores of health related quality of life with interviewer administrated than self-administrated versions. Whether health-related quality-of-life measurements are affected by the format of administration or not, is a subject of discussion [31, 37, 38].

Some items in NEI-VFQ 25 needed more explanation, probably mostly due to an aging population with various degree of hearing impairment. With the combination of high age and reduced central vision, we thus recommend the interviewer-administered format of NEI-VFQ 25 as preferable for nAMD patients. Our study population of Norwegian nAMD patients had higher sub-scores and overall score of NEI-VFQ 25 at baseline (79.5), compared to the two large studies of nAMD populations MARINA (69.3) and ANCHOR (69.9) [19]. The only lower sub-score in our study compared to MARINA and ANCHOR was general health. The high overall score at baseline may be a reflection of the efficiency in the early diagnosis of nAMD in the present patient population of the Norwegian capital and surrounding areas. One explanation for lower general health sub-score of this nAMD Norwegian population might be due to the patient’s high age (83.4 years) compared to MARINA (77.1) and ANCHOR studies (77.0 years).

High face validity in our study suggests that NEI-VFQ 25 can be considered as relevant by the patients. This can be due to the patient-derived way NEI-VFQ was originally developed by including patients to identify problems in different visual diseases like AMD [39].

Our results show that all correlations are below 0.5, hence 0.5^2^ = 25% or less of the variations are explained by the visual acuity for treated eye and best eye, EQ-5D and PASS 5 (in Table 3) [40]. These moderate correlations were similar to those found by two other studies performed on nAMD population [20, 27]. NEI-VFQ 25 validity seems acceptable, since it is significantly correlated to most relevant variables. However, a possible explanation for the generic EQ-5D not significantly being associated to the disease specific NEI-VFQ 25 at 6 and 12 months may be that disease specific measures are more sensitive to change, and that general health will usually not change as quick as vision in nAMD patients.

Concerning the associations between sub-scores and objective visual acuity, our results are similar to other studies showing strongest correlations between the sub-scores and best eye, confirming that patients who have a lower best corrected visual acuity report lower visual function. However, preserving vision on treated eye indicates a significant impact on visual function [41–43]. Sub-categories as peripheral vision, ocular pain and general health were not significantly correlated with visual acuity nor with PASS 5, which may indicate that these sub-scores are not the strongest associates for patients with nAMD which is coherent with other studies on same population [5, 41]. Orr et al., who were examining correlations between sub-scores and visual acuity in best and worst eye, found similar results (except for peripheral vision) [20]. Results were also similar in the same population with the longer version of NEI-VFQ 39 [42].

Testing reproducibility with test-retest at 6 months after initiation of treatment was chosen since clinical experience has shown that this period is more stable for the patients than earlier in treatment. A possible limitation of this test is the risk of patients remembering the questions of NEI-VFQ 25, since the set time limit between the test and retest was only one week. However, shorter period like this for a relatively large sample size, 25 questions and an older patient population who easily forget, are likely to give memory minor influences on the retest.

High reliability was demonstrated in test-retest analyses for patient group, showing the majority (9 of 12) of the sub-scores with a good or excellent ICC values and 3 out of 12 being moderate. The overall score had excellent reliability (0.91) [30]. Other studies testing NEI-VFQ 25 on different ocular diseases including nAMD have shown ICC values from 0.6–0.7 and higher (except for general vision and role difficulties) [13, 16, 17, 44]. Lower ICC values have been shown in other studies on general vision and general health [45, 46]. Some values in test-retest analyses for the healthy controls were reported to be poor in our study, such as color vision, which could be due smaller sample and the potential ceiling effect [47]. Regarding internal consistency, Sorensen et al., reported a low value of 0.4 on ocular pain and 0.5 on driving [17]. Other studies have reported similar results in nAMD populations, which are coherent to our results [20, 42].

We found a significant difference between the patients and controls which supports discriminatory power, both for the overall score and most sub scores – excluding color- and peripheral vision and ocular pain, which is in agreement with the results from the Danish study [17]. Another study performed on patients with nAMD showed significantly lower scores than control group for all sub-scores except for ocular pain [36]. The discriminatory power of NEI-VFQ 25 has also been shown in other studies of ocular diseases including nAMD [15, 16, 45, 46]. Our results support that the Norwegian version of NEI-VFQ 25 is responsive, which is coherent with the results from a large study on a similar population [19]. These results are coherent to statistically significant improvement in objectively observed visual acuity by LogMar both on treated and best eye. Responsiveness has also been tested in other studies exploring psychometric properties of NEI-VFQ 25 on different ocular diseases including nAMD and has overall shown similar results [45, 48]. Miskala et al. found that peripheral and color vision and ocular pain were not significantly affected by poor vision and less affected in nAMD [21], which is similar to our results. Other studies have also found corresponding correlations between sub-scores and visual acuity, which are associated with central vision [15, 44, 48].

Rasch analyses and Item Response Theory are relatively new analyses used to develop, evaluate and improve patient reported outcomes. They might have been relevant in current study, to both evaluate and potentially suggest improvement in items of NEI-VFQ 25 as an previous study has shown in the same population [49]. However, the aim in the present study was not such an extensive evaluation, but rather to validate the original measure in a Norwegian population with nAMD. We believe that this would be a valuable approach in future evaluations of NEI-VFQ-25 in this population.

## Conclusion

The Norwegian version of NEI-VFQ 25 showed satisfactory psychometric properties, including validity, reliability, responsiveness, discriminatory power and predictive value. The added value of monitoring patient’s subjective visual function in clinical settings, is a better understanding of how to target the individualized needs of the patients with nAMD. Acknowledging the requirement for psychometric performance of PROM that is shown in this study, may support an extended use of NEI-VFQ 25 in patients with nAMD in scientific studies in Norway.

## Data Availability

The dataset generated during the current study are not publicly available, however are available from the corresponding author on reasonable request.

## Abbreviations

anti-VEGF: anti–vascular endothelial growth factor
EMA: The European Medicines Agency
EQ-5D-3 L: EuroQoL Health Questionnaire
FDA: The Food and Drug Administration
ICC: Intraclass Correlation Coefficient
LogMar: Logarithm of the minimal angle of resolution
nAMD: neovascular Age-related Macular Degeneration
NEI-VFQ 25: National Eye Institute Visual Function Questionnaire
OCT: Optical Coherence Tomography
PASS 5: Patient Acceptable Symptom Status
PROM: Patient reported outcome measures
T/E: Treat and Extend
